# Evaluation of total intravenous anesthesia with remimazolam in general anesthesia for pulmonary endarterectomy of chronic thromboembolic pulmonary hypertension: a case report

**DOI:** 10.1186/s40981-023-00626-8

**Published:** 2023-06-12

**Authors:** Sae Igarashi, Yusuke Ishida, Shunya Sekiguchi, Yosuke Fujita, Aya Kawachi, Mikiko Tomino

**Affiliations:** Department of Anesthesiology, Tokyo Medical University, 6-7-1 Nishishinjuku, Shinjuku-ku, Tokyo, 160-0023 Japan

**Keywords:** Remimazolam, Pulmonary endarterectomy (PEA), Chronic thromboembolic pulmonary hypertension (CTEPH), Pulmonary vascular resistance (PVR)

## Abstract

**Background:**

Pulmonary endarterectomy (PEA) is a treatment modality for chronic thromboembolic pulmonary hypertension (CTEPH). PEA requires anesthesia management to prevent an increase in pulmonary vascular resistance (PVR) and circulatory failure. Therefore, it is necessary to select an anesthetic agent that can achieve these goals as much as possible. On the other hand, remimazolam, a short-acting sedative, was launched in Japan in 2020, and its use in various cases has been increasingly reported. This report demonstrates that remimazolam can be used safely in the anesthetic management of PEA.

**Case presentation:**

A 57-year-old man was scheduled to undergo PEA for CTEPH. Remimazolam was used for sedation from induction of anesthesia. Hemodynamics were stable during surgery without circulatory failure. Anesthesia was managed intraoperatively without any particular increase in PVR.

**Discussion:**

Anesthesia was successfully managed without any complications. This case suggests that remimazolam is one of the options for anesthetic management in PEA.

## Background

In the anesthetic management of pulmonary endarterectomy (PEA) for chronic thromboembolic pulmonary hypertension (CTEPH), it is crucial to avoid overloading the right heart and to minimize the increase in pulmonary vascular resistance (PVR). Remimazolam, a short-acting benzodiazepine, has been available in Japan since 2020 [1]; it has the advantage of less circulatory suppression [2]. The successful anesthetic management of PEA using remimazolam is reported. Written, informed consent was obtained from the patient to publish this case report.

## Case presentation

A 57-year-old man (height: 167 cm; weight: 76 kg) noticed dyspnea on light exertion about 1 year and 6 months earlier, and his symptoms gradually worsened. About 1 year earlier, he visited a nearby hospital and was diagnosed with CTEPH by contrast-enhanced computed tomography (CT) and echocardiography. Acute treatment with heparin was administered at the local clinic, and treatment was continued with edoxaban 60 mg/day. He was then admitted to our hospital for PEA to further improve his exercise capacity. His medical and family histories were unremarkable.

His findings were as follows: New York Heart Association functional class II; blood pressure 118/74 mmHg; heart rate 87 beats/min; and peripheral blood oxygen saturation (SpO_2_) 95% (room air), decreased to 88% (room air) on a 6-min walk test. Right heart catheterization showed pulmonary arterial pressure (PAP) 47/14 (26) mmHg, pulmonary capillary wedge pressure 9 mmHg, cardiac output 5.12 L/min, cardiac index 2.65 L/min/m^2^, PVR 249 dynes·s/cm^5^, and right atrial pressure 5 mmHg. Transthoracic echocardiography showed a left ventricular ejection fraction of 64%, an end-diastolic dimension/end-systolic dimension of 58/37 mm, local contraction abnormalities of ventricular wall motion, a tricuspid annular plane systolic excursion of 20 mm, mild tricuspid regurgitation, mild mitral regurgitation, and no interventricular septal pressure.

**Fig. 1:**
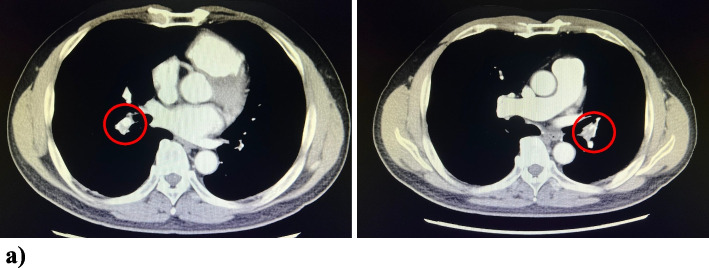
Thoracic contrast-enhanced computed tomography. **a** Pulmonary thrombi are found in the pulmonary arteries on both sides (red circle)

Thoracic contrast-enhanced CT showed chronic thrombosis in the lingual segment and left lower lobe branches, as well as in the middle and right lower lobe branches, with some stenosis of the pulmonary arteries (Fig. 1). Pulmonary perfusion scintigraphy showed decreased blood flow in both lower lungs and in the left pulmonary lingual segment.

**Fig. 2:**
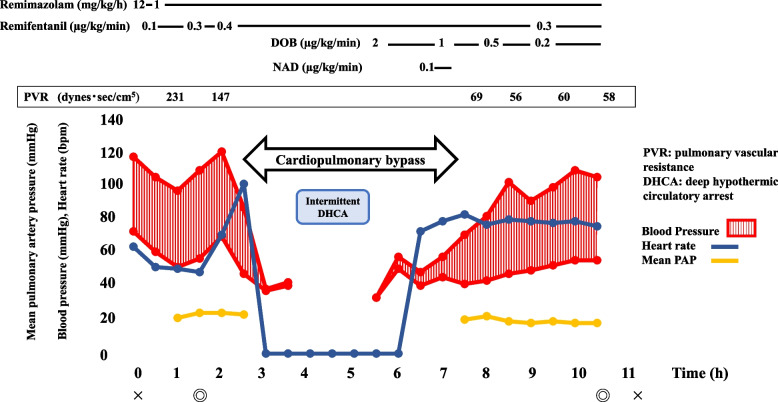
Anesthetic record

Anesthesia was induced with remimazolam 12 mg/kg/h, fentanyl 500 µg, remifentanil 0.1 µg/kg/min, and rocuronium 70 mg, and it was maintained with remimazolam 1 mg/kg/h, remifentanil 0.3–0.4 µg/kg/min, and rocuronium 30 mg/h. The trachea was intubated with an 8.0-mm-ID endotracheal tube. When blood pressure decreased, 0.05–0.1 mg of phenylephrine was administered as needed. Invasive arterial pressure, SpO_2_, PAP, central venous pressure, the Patient State Index (PSI), regional oxygen saturation (rSO_2_), and transesophageal echocardiography were monitored during surgery. PAP was monitored continuously by a Swan-Ganz catheter with a Hemosphere monitor (Edwards Lifesciences Co., Tokyo, Japan). During the induction period, the mean PAP and PVR remained at approximately 30 mmHg and around 150 dynes·s/cm^5^, respectively (Fig. 2). Siverestat was administered at 12 mg/h from the start of induction for lung protection. After median sternotomy, the patient was placed on cardiopulmonary bypass (CPB) without any significant circulatory disturbances, and PEA was performed under deep hypothermic circulatory arrest (DHCA). PEA of bilateral pulmonary arteries was performed. During intermittent DHCA (body temperature of 18 °C), PSI was 0 due to hypothermia and was not evaluable. Dobutamine 1.3 µg/kg/min and norepinephrine 0.1 µg/kg/min were administered continuously during weaning from CPB. Norepinephrine was used only at the time of weaning from CPB and was discontinued immediately. After weaning from CPB, his hemodynamics remained stable. Therefore, the anesthesia was well managed. The dose of dobutamine was gradually reduced to approximately 0.2 µg/kg/min at the end of surgery. Respiratory management was performed with a positive end-expiratory pressure of 10 cmH_2_O from the time of weaning from CPB (ventilation was maintained at 6–8 mL/kg of ventilation with a pressure-controlled ventilation set at 40% fraction of inspiratory oxygen). Remimazolam was continued at 1 mg/kg/h after weaning from CPB. The PSI, which was used as an index of sedation, remained at approximately 20 to 30.

Anesthesia time was 11 h 10 min, operation time was 8 h 51 min, CPB time was 4 h 44 min, and circulatory arrest time was 1 h 16 min. Total transfusion volume was 3165 mL, urine volume was 1890 mL, and blood loss was 2546 mL. The patient received transfusion of 6 units of red blood cell concentrate, 8 units of fresh frozen plasma, and 1000 mL of 5% albumin product. After admission to the intensive care unit, the patient was weaned from mechanical ventilation and extubated on postoperative day (POD) 1. He was transferred to the general ward on POD 3. On POD 19, PAP was 22/3 (11) mmHg, and PVR decreased to 106.7 dynes·s/cm^5^. The patient did well and was discharged on POD 20.

## Discussion

CTEPH is a progressive disease with significant morbidity and mortality. It has a poor prognosis due to the limited efficacy of medical therapy, and PTE is considered an effective treatment [3]. PEA is a procedure performed under very hypothermic (16–18 °C) intermittent circulatory arrest. The left and right main pulmonary arteries are incised, an appropriate dissection surface between the internal elastic plate and tunica media is found, and then one proceeds to the regional artery and removes the organizing thrombus [4]. However, PEA is not indicated in patients presenting with heart failure because they may not be able to maintain their circulatory status; therefore, transcatheter therapy or pharmacotherapy is more likely to be used [5]. In addition, since preoperative right heart function is thought to affect the prognosis of PEA, caution is advised in patients with high levels of brain natriuretic peptide (BNP), an index of right heart function [6].

Higher levels of PVR and PAP can worsen the outcome of PEA [7], and it is critical to minimize the increases in PAP and PVR. Anesthetic management is also required to ensure that the right heart is not stressed. In the present case, the PVR was 249 dynes·s/cm^5^, and the BNP was below the lower limit of measurement; because the PVR was not very high and the BNP was low, the patient appeared to have relatively mild CTEPH. However, there was a possibility that right heart failure was caused by the long-term stress on the right heart, and caution was required during induction of anesthesia. Therefore, to avoid circulatory collapse during induction of anesthesia, it is necessary to select sedatives with less circulatory depression and to pay attention to the dosage of these drugs. The same applies to the administration of analgesics.

Remimazolam, a short-acting benzodiazepine general anesthetic, was recently launched in Japan [1]. Like propofol, it has a rapid onset of action and inactive metabolites, allowing assessment of unconsciousness by electroencephalography [8, 9]. It also has a shorter half-life than midazolam. Unlike propofol, water-soluble remimazolam does not contain lipids in its solvent, results in no lipid loading during long-term administration, and the risk of bacterial growth appears to be low. Moreover, it has the clinical advantage of not causing vascular pain, unlike propofol [9]. Prior to the launch of remimazolam, propofol was typically chosen for intraoperative general intravenous anesthesia, but it has a strong circulatory inhibitory effect [10], which makes it difficult to use in patients with severe right heart failure. On the other hand, remimazolam is a short-acting benzodiazepine used for general anesthesia. It has less circulatory depression and provides stable circulatory dynamics even in patients with cardiac insufficiency [11–13]. It was also reported that midazolam, a benzodiazepine, had little effect on PVR [14], and remimazolam, also a benzodiazepine, may have little effect on PVR. In the present case, both PAP and PVR remained stable and could be managed without any circulatory disturbance. Common immediate postoperative complications of PEA are reperfusion lung injury and intratracheal hemorrhage, which require attention [4]. On searching PubMed for case reports of anesthetic management with remimazolam for PEA in patients with CTEPH, no such report was found. Therefore, the present experience with this case demonstrates for the first time that remimazolam is effective in the anesthetic management of patients with CTEPH. In addition, remimazolam has the characteristic of less circulatory depression; therefore, it may become a standard anesthetic in the future [2]. PEA is characterized by intermittent circulatory arrest for 10 to 15 min during cardiopulmonary bypass [15]. Although there have been previous reports of the safe use of remimazolam in surgeries involving cardiopulmonary bypass [16], there have yet been no reports of cardiopulmonary bypass with DHCA using remimazolam. The present case report demonstrates that remimazolam could be safely used without causing central nervous system or other complications due to DHCA.

## Data Availability

Not applicable.

## Abbreviations

PEA: Pulmonary endarterectomy
CTEPH: Chronic thromboembolic pulmonary hypertension
PVR: Pulmonary vascular resistance
CT: Computed tomography
SpO_2_: Peripheral blood oxygen saturation
PAP: Pulmonary arterial pressure
PSI: Patient State Index
rSO_2_: Regional oxygen saturation
CPB: Cardiopulmonary bypass
DHCA: Deep hypothermic circulatory arrest
POD: Postoperative day
BNP: Brain natriuretic peptide
